# A 3D printable adapter for solid-state fluorescence measurements: the case of an immobilized enzymatic bioreceptor for organophosphate pesticides detection

**DOI:** 10.1007/s00216-021-03835-1

**Published:** 2022-01-22

**Authors:** Andreia C. M. Rodrigues, Maria Vittoria Barbieri, Marco Chino, Giuseppe Manco, Ferdinando Febbraio

**Affiliations:** 1 Institute of Biochemistry and Cell Biology, National Research Council (CNR), 80131 Naples, Italy; 2 Department of Chemical Sciences, University of Naples “Federico II”, 80126 Naples, Italy

**Keywords:** Fluorescence/luminescence, Pesticides/endocrine disruptors, Biosensors, 3D printed adapter, Esterase-2, Membrane support

## Abstract

The widespread use of pesticides in the last decades and their accumulation into the environment gave rise to major environmental and human health concerns. To address this topic, the scientific community pointed out the need to develop methodologies to detect and measure the presence of pesticides in different matrices. Biosensors have been recently explored as fast, easy, and sensitive methods for direct organophosphate pesticides monitoring. Thus, the present work aimed at designing and testing a 3D printed adapter useful on different equipment, and a membrane support to immobilize the esterase-2 from *Alicyclobacillus acidocaldarius* (EST2) bioreceptor. The latter is labelled with the IAEDANS, a bright fluorescent probe. EST2 was selected since it shows a high specificity toward paraoxon. Our results showed good stability and replicability, with an increasing linear fluorescent intensity recorded from 15 to 150 pmol of labelled EST2. Linearity of data was also observed when using the immobilized labelled EST2 to detect increasing amounts of paraoxon, with a limit of detection (LOD) of 0.09 pmol. This LOD value reveals the high sensitivity of our membrane support when mounted on the 3D adapter, comparable to modern methods using robotic workstations. Notably, the use of an independent support significantly simplified the manipulation of the membrane during experimental procedures and enabled it to match the specificities of different systems. In sum, this work emphasizes the advantages of using 3D printed accessories adapted to respond to the newest research needs.

**Graphical abstract:**

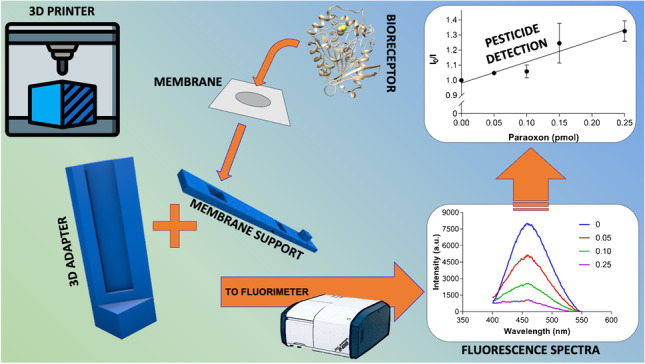

**Supplementary Information:**

The online version contains supplementary material available at 10.1007/s00216-021-03835-1.

## Introduction

Organophosphate pesticides (OPs) are a group of chemicals that irreversibly inhibit the activity of acetylcholinesterase (AChE), an enzyme responsible for the transmission of the synaptic nerve impulse in the central and peripheral nervous system [1]. OPs have been widely used as insecticides, fungicides, and herbicides in the last decades in agricultural pest control, and nowadays, they are among the most commonly used classes of pesticides worldwide [2, 3]. Although OPs are meant to eradicate specific pests, their high application has led to their accumulation in the environment, contaminating water resources, soils, and foods (vegetables, fruits) and affecting the health of non-target organisms, including biota and human beings [4, 5]. A few simple catalysts are emerging for environmental remediation [6–9]. However, their application is still far to come, being urged to identify contaminated areas and feedstocks.

Several methodologies have been developed to analyse and detect OPs in specific matrices, considering OPs properties and toxicological effects. Traditional techniques include gas chromatography (GC) or liquid chromatography (LC) coupled to mass spectrometry (MS) [10–15], which present the advantage of achieving very high sensitivity and selectivity, allowing to obtain accurate OPs determination. Nevertheless, these methodologies are time-consuming and costly, require pretreatment processes and the need of skilled professionals, and are not suitable for direct and continuous monitoring of pesticides in the field. Recently, fast, easy, and cheap methodologies have been successfully developed using different fluorescent biosensors to detect pesticides in environmental matrices [16–20]. The majority of biosensors are based on the inhibition of acetylcholinesterase activity [21–25]. However, these systems present low stability after changes in environmental conditions and low specificity toward inhibitor compounds, possibly reacting with other compounds than OPs. In our previous works [19, 20, 26, 27], esterase-2 from *Alicyclobacillus acidocaldarius* (EST2) was used as an alternative biosensor for OP detection. This enzyme shows a very high affinity toward paraoxon (SI Fig. S1), interacting similarly to the substrate, and forming a covalent intermediate with the catalytic Ser155 in the enzyme active site [18]. However, the binding is too stable to be dissociated with the water molecule acting as nucleophile, producing a stable covalent intermediate, which irreversibly inactivates the enzyme according to a high affinity irreversible inhibition [28]. EST2 specificity and sensitivity led to a calculated limit of detection (LOD) in the nanomolar range of paraoxon. As recently demonstrated, EST2 can also recognize other OPs [20, 27]; however, the oxidation of phosphorothionate OPs by using a strong oxidant, such as N‐bromosuccinimide, is needed prior to its covalent binding to the protein [27]. In fact, similarly to acetylcholinesterases, EST2 shows lower affinity toward phosphorothionate OPs, although it seems to be able to reversibly bind some of them, such as parathion and chlorpyrifos [18, 19].

Moreover, being a thermostable enzyme, EST2 shows high stability toward temperature, maintaining its activity for weeks at room temperature after immobilization, pH variations, and organic solvents [26].

The development of enzyme-based biosensors is currently an important subject of research since they offer the advantage, over conventional methodologies, to be used on-site and in real-time monitoring analysis [17, 19, 29, 30], of great interest, especially for their application in environmental and food contamination measurements. Nonetheless, real samples often contain organic substances such as pigments, vitamins, and amino acids, which manifest an intrinsic fluorescence which may interfere with fluorescent measurements [31]. The immobilization of the enzymes to a solid surface can be a simple and effective way of avoiding such interference. This procedure has the advantage of maintaining the enzyme activity over time in a support that, on its own, exhibits biocompatibility keeping the enzyme bioactivity and stability [32].

Different immobilization materials have been used for the development of enzyme-based biosensors, including self-assembled multilayer (SAMs) [32], eggshell membrane (ESM) [33], and nanomaterials (e.g. nanoparticles, quantum dots, carbon dots, among others) [34]. In this work, we selected a polyvinylidene difluoride (PVDF) membrane. This naturally hydrophobic fluoropolymer membrane exhibits hydrophobic interactions with a wide range of proteins, offering the advantage of high binding capacity and long-term stability of immobilized proteins [35, 36]. Notwithstanding, fluorometric analysis of proteins in the immobilized form is not easy to perform, considering that usually, standard cuvette sample holders are used for routine protein analysis in liquid samples and are not designed for immobilized protein. The adapters for the spectrofluorometer systems commercially available are expensive and sometimes do not cover the specific needs of new forefront experiments [37]. Moreover, these accessories require a specific design to guarantee the performance of the UV–visible spectrometer. In fact, the full spectra transparency is not achieved using materials such as transparent polypropylene or polylactic acid (PLA) [38].

In light of this, this work aimed at developing an immobilized enzymatic biosensor by using a 3D printed adapter to perform fluorescence measurements. A cuvette adapter and support for the solid membrane were printed using a high-precision mini 3D printer. This machine uses filamentous thermoplastic material to draw three-dimensional objects in a user-friendly, fast, and configurable way. The design of the adapter was optimized regarding its shape, dimension, and angle to provide versatility for its use in different systems, with minor design adjustments. The applicability of the immobilized EST2 as bioreceptor for OPs monitoring was evaluated using the organophosphate paraoxon.

## Materials and methods

### Reagents

All reagents were of analytical grade and obtained from commercial sources. Diethyl-p-nitrophenyl phosphate (paraoxon), tributylphosphine (TBP), tris(hydroxymethyl)aminomethane hydrochloride (Tris/HCl), and dimethyl sulfoxide (DMSO) were purchased from Sigma-Aldrich (Merck & Co., Inc., USA). Bio-Rad dye reagent was purchased from Bio-Rad Laboratories, Inc, USA. 5-((((2-Iodoacetyl)amino)ethyl)amino) naphthalene-1-sulfonic acid (1,5-IAEDANS) was purchased from Molecular Probe (Thermo Fisher Scientific, USA).

### Over-expression and purification of EST2-S35C in *Escherichia coli*

EST2-S35C was over-expressed in the mesophilic host *E. coli* strain BL21 (DE3), extracted and purified as previously described in Carullo et al. [19], with slight modifications. This strain has been early developed in our laboratory [39]. As previously described, we added TBP, a reducing agent, to the solutions to preserve the mutated cysteine in the reduced form. The protein was extracted by using a sonication step performed on a Branson Sonifier Sound Enclosure Model SSE-1 instrument using 3 cycles of 40 s ON/30 s OFF pulses at 50% power output intensity in a water/ice bath at 4 °C. The cell debris was removed by ultracentrifugation (80,000* g* at 4 °C for 30 min). After the thermo-precipitation steps, purity of the enzyme > 95% was reached by a gel filtration step using a Sephadex G-25 column (GE Healthcare Bio-Sciences AB, Sweden). The protein concentration was estimated according to the Bradford method [40], with bovine ɣ-globulin as the standard.

### Binding probe-enzyme

IAEDANS was dissolved in DMSO, as a polar solvent, at the final concentration of 20 mM. As described in [19], EST2-S35C (15 × 10^−9^ mol), in 25 mM Tris/HCl buffer at pH 7.5 and 1 mM TBP, as reducing agent, was conjugated with IAEDANS (tenfold molar excess), by incubating overnight in the dark at room temperature. The excess of the probe was removed by dialysis against a 25 mM Tris/HCl buffer at pH 7.5, at room temperature in the dark by using a quicksep adapter. The protein-probe concentration was determined using the Bio-Rad dye reagent, as described by Bradford et al. [40].

### Protein immobilization

The protein was immobilized on a PVDF polyvinylidene difluoride (PVDF) hydrophobic fluoropolymer membrane (pore size 0.20 µm) (PORABLOT—MACHEREY–NAGEL GmbH & Co. KG, Germany), a high-quality transfer membrane for biomolecules. The membrane was cut to the desired size, activated in 100% ethanol for 5 min fully submerged, washed with distilled water, and transferred in a 25 mM Tris/HCl buffer at pH 7.5 for 5 min to equilibrate. After that, the excess of Tris/HCl was removed, and 5 µl protein at the desired concentrations was spotted on the membrane surface and let to be physically adsorbed by drying at room temperature in the dark.

### 3D adapter design

The 3D adapter (12 × 12 × 45 mm) and support (8 × 3 × 36 mm) for the membrane were designed using the software SketchUp Make 2017 (Trimble Inc., USA). Slicing was done using the software Ultimate Cura v4.9.1.1 (Ultimaker B.V., Netherlands), and printing parameters were established on Labslicer 3D Slicing Software for Windows (Labists, Hongyu Zhineng Technology Co., Ltd., China). Prints were made at a nozzle temperature of 180 °C, a print speed of 30 mm/s with 80% infill density, and 20 mm/s with 100% infill density for the adapter and membrane support, respectively. To avoid light refraction, black polylactic acid (PLA) 1.75 mm filament was used to print both components in the High Precision Mini 3D printer, X1 entry-level 3D printer DIY kit, from Labists. Previous adapters were also printed, testing for the angle of light incidence and reflection, and the need to use the membrane support holder. The digital designs are available free of charge under a creative commons licence (https://www.thingiverse.com/febbraio-research-group/designs).

### Fluorescence measurements

A stock solution of 1.0 µg/µl (~ 30 pmol) EST2-S35C labelled with IEADANS was used to spot onto the membranes. Fluorescence measurements of the labelled proteins immobilized on the PVDF support were performed, adding to the membranes different amounts of labelled EST2-S35C (0, 15, 30, 45, 90, and 150 pmol) in a final volume of 5 µl (previously defined as the best volume to spot on the area of the membrane used). The fluorescence of the membranes was measured after washing with 25 mM Tris/HCl buffer, pH 7.5.

Measurements of the fluorescence quenching for the EST2-S35C inhibition assays were performed, adding increasing concentrations of OPs to the membranes. A stock solution of paraoxon (10 mM) in DMSO was prepared, and increasing amounts of paraoxon (0, 11.7, 23.4, 35.1, 46.8, 58.5, 117.0, 175.5, 234.0 pmol) were added (5 µl) to the immobilized labelled EST2-S35C. The membranes were incubated for 1 min, briefly washed with 25 mM Tris/HCl buffer, pH 7.5, and the emission spectra were acquired.

Fluorescence spectroscopy measurements were carried out in a Jasco FP-8200 (JASCO, Tokyo, Japan) and a Horiba Jobin–Yvon Fluoromax-4 (HORIBA Northampton, UK) spectrofluorometers. The emission spectra of the fluorescent probe conjugated with EST2-S35C were recorded in the range from 380 and 550 nm using an excitation wavelength of 340 nm, 1 nm step resolution, and 500 nm/min scan speed with 3 accumulations. In the case of the unlabelled EST2-S35C, an excitation wavelength of 280 nm was used, and emission spectra were recorded from 300 to 500 nm, with 1 nm step resolution, and 500 nm/min scan speed with 3 accumulations. Unless otherwise stated, the readings were performed in triplicate and three membranes per protein amount were used.

The developed 3D adapter was used as support for the PVDF membrane for the detection of immobilized protein. Spectra Manager 2.09 software from JASCO and FluorEssence™ software from Horiba were used for instrument control and data acquisition.

### Data analysis

The linearity of data was assessed, and the robustness of replicates was tested by *F* test with *P* < 0.05. Limit of detection (LOD) and quantification (LOQ) were calculated using the equations LOD = 3*(SD/slope) and LOQ = 10*(SD/slope), respectively. Statistical analysis was performed using the software GraphPad Prism version 7.0 for Microsoft Windows (GraphPad Software, La Jolla, CA, USA).

## Results and discussion

### 3D adapter design and optimization

The adapter structure was designed in a similar shape and dimension of a traditional cuvette, fitting the cuvette holders, thus providing the versatility for its use with different fluorometers (SI Fig. S2). Both white and black PLA were tested, and the biodegradable black PLA was selected as it minimizes the refraction of light. The suitability of the black printable material to obtain cuvette adapters for fluorescent measurements has also been previously shown [37].

**Fig. 1:**
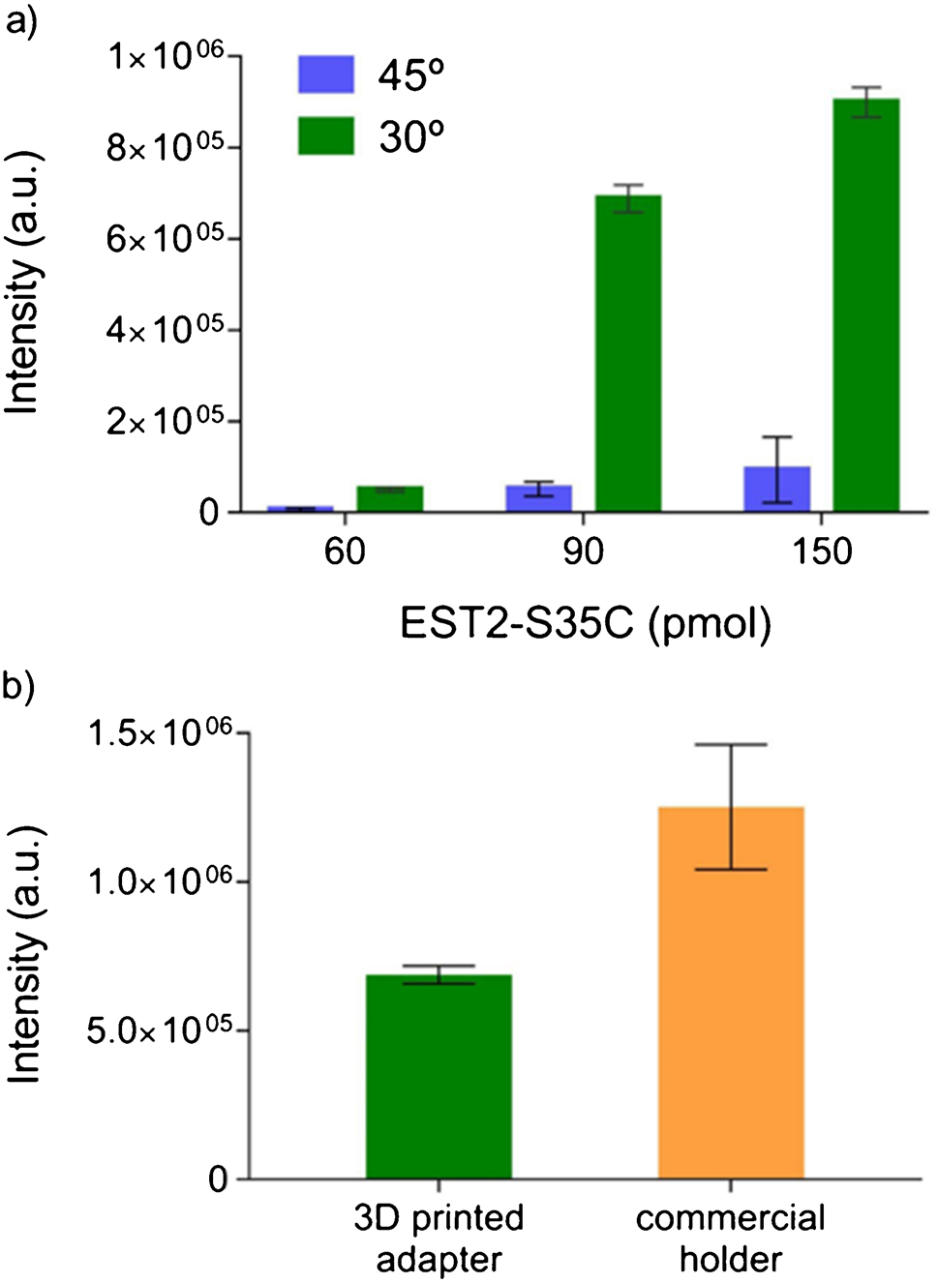
(a) Fluorescence intensity as a function of protein concentration and angle of incident light (45° blue bars, 30° green bars). (b) Fluorescence intensity comparison between the 3D printed (green) and Horiba proprietary (orange) systems. Data is presented as mean ± standard deviation (SD), *n* = 3

A pilot version of the adapter was designed for an incident beam angle of 45°. However, this resulted in low signal/noise ratios, and increased standard deviation for the fluorescence intensity and maximum emission wavelength (Fig. 1a, SI Table S1). Therefore, the incident angle was adjusted to 30° in the final/optimized version, which improved the emission signal, by decreasing the extent of scattering of the incoming light at the excitation wavelength (Fig. 1a, SI Fig. S2).

**Fig. 2:**
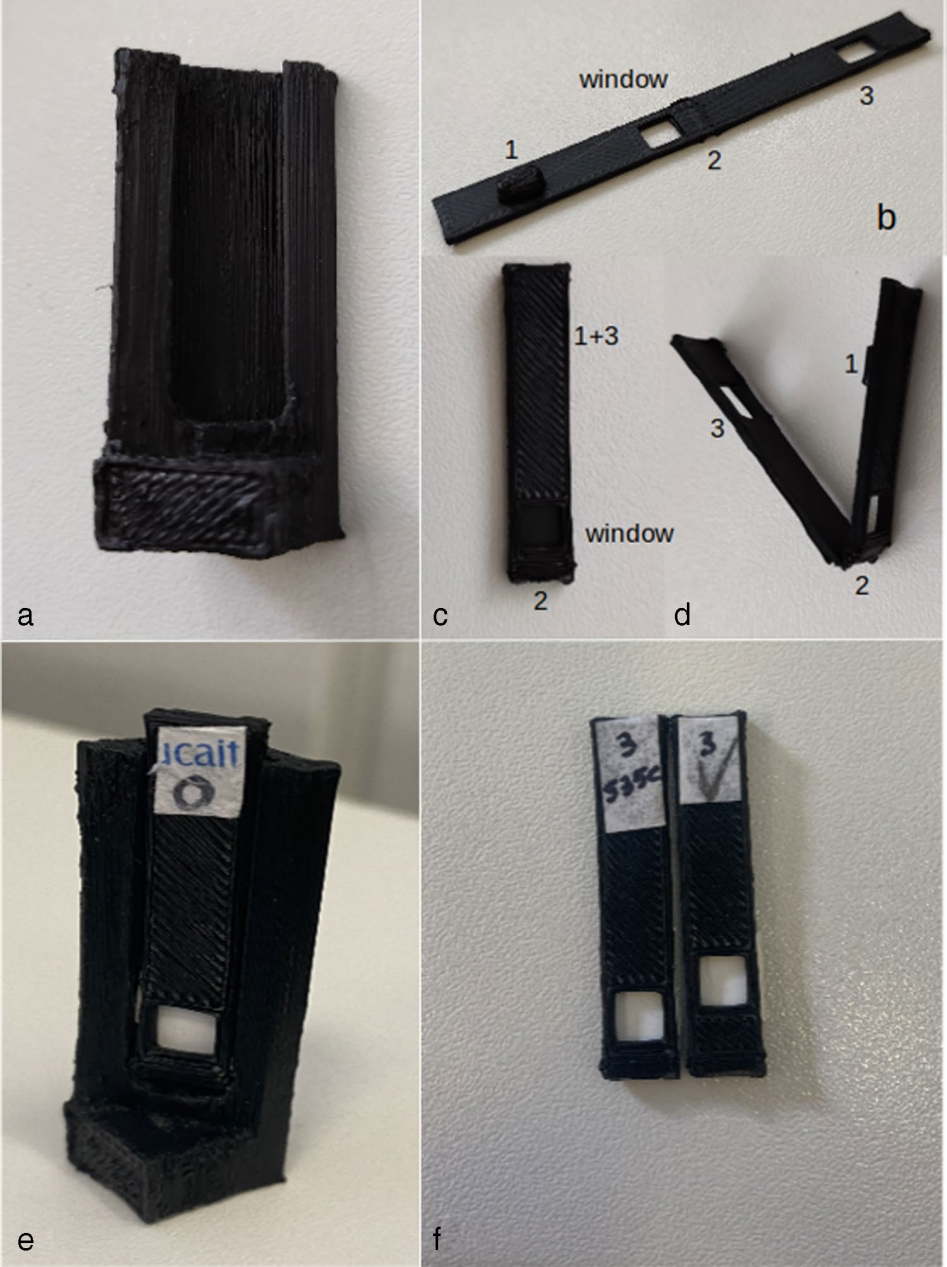
General illustration of the 3D printed adapter and membrane support: (a) frontal view of the 3D adapter; (b) printed support, (c) close configuration from the front, and (d) open configuration from the side, with the indication of the window for the placement of the membrane, (2) the fold and (1, 3) the system for closing the support; (e) assembled 3D adapter with the membrane placed in the support; (f) supports with different window heights for the use in different spectrofluorometers

The development of a specific support assured the stability and the accuracy of the membrane location into the adapter (Fig. 2a–d). This support was designed to fit the 3D printed adapter (Fig. 2e) and carries a small window where the membrane is allocated and blocked before the spotting of the protein. This system enables the protein spotting in the same position among different membranes, ensuring replicability. The ease in membrane manipulation represents another advantage of the support, facilitating the necessary experimental procedures (wash, addition of the sample) without touching/perturbing the membrane. Furthermore, the height of the window on the support was optimized to maximize the light beam intensity in the centre of the membrane (SI Table S2). Nevertheless, the most suitable window height of the support may be different among different instruments. Therefore, we provide a set of membrane supports at different window heights, compatible with different fluorometer systems (Fig. 2f). A spectrum of immobilized EST2-S35C was also obtained using the commercially available holder for solid samples on Horiba Fluoromax-4 (Fig. 1b, SI Fig. S3 and S4). Adopting the same instrumental parameters, despite being stronger than our printed apparatus, the fluorescence signal intensity with the commercial holder was so high that it almost saturated the detector. Finally, our support perfectly fits most common cell holder and Peltier units, thus allowing the user to perform temperature-controlled experiments without wasting time performing complex installation procedures of commercial sample holders.

Preliminary trials were performed to define the optimal volume and concentration of bioreceptor to be used by testing different volumes of the protein spot (0.5, 1, 2, 4, and 6 µl, SI Table S3). The final volume of 5 µl containing a more diluted concentration at 90 pmol of labelled protein was selected because it granted a homogeneous distribution of the protein onto the membrane and a better fluorescence intensity.

### EST2-S35C immobilization on membrane

Exploiting the ability of esterases, and in particular of EST2, to strongly and easily bind to surfaces by hydrophilic/hydrophobic interactions, we immobilized the protein by physical adsorption on a PVDF hydrophobic membrane. The physical adsorption strongly reduces the influence of the matrix on the enzyme activity, being not involved in covalent binding that could stretch the macromolecule or occupy the catalytic site. After drying, the binding of EST2 with the membrane becomes very stable and it is retained after extensive washing; moreover, the spot is not observed to spread over time as demonstrated by fluorescence measurements.

### Fluorescence analysis of EST2-S35C

**Fig. 3:**
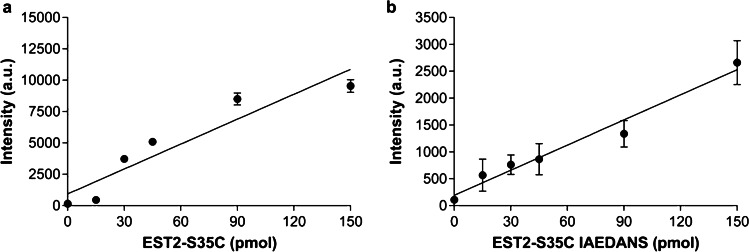
Fluorescence intensity (mean ± SD, arbitrary units, *n* = 3) for increasing amounts of immobilized (a) EST2-S35C (pmol) at emission wavelength 331–335 nm, and (b) EST2-S35C labelled with IAEDANS (pmol) at emission wavelength 463–474 nm

To test the performance of the 3D printed adapter and membrane support, we evaluated the fluorescence of the EST2-S35C protein under different conditions. In particular, we measured the intrinsic and extrinsic fluorescence after protein labelling. The intensity of tryptophan from the immobilized EST2-S35C was evaluated, and a significant linear relationship (*Y* = 66.24*X* + 938.8, *r*^2^ = 0.88, *Sy.x* = 1514, *F*_1,4_ = 29.85, *P* = 0.006) was observed with increasing protein amount (Fig. 3a).

Good replicability and linear relationship between the intensity of the fluorescent signal and the amount of immobilized labelled protein were also observed in the case of IAEDANS probe. In this case, three different membranes were tested for each protein amount and no significant differences were observed among the slopes and the intercepts of the three replicates. Therefore, it was possible to calculate a unique equation to describe the linear increase in fluorescence with increasing protein amount: *Y* = 15.59 *X* + 192.8, *r*^2^ = 0.97, *Sy.x* = 175, *F*_1,4_ = 123.8, *P* = 0.0004 (Fig. 3b).

As expected, our results highlight the advantage of using a fluorescent probe, such as IAEDANS, instead of measuring only the intrinsic fluorescence of tryptophan, allowing to obtain better sensitivity even at lower amounts of protein.

### Application of the biosensor for organophosphates detection

Fluorescence spectroscopy is among the most used techniques in analytical chemistry laboratories, mainly due to their high sensitivity and simplicity. Detection of pesticides in environmental samples is one of the current challenges that requires great sensitivity. Therefore, paraoxon was used as a model OP to evaluate the efficacy of our immobilized bioreceptor, given the high affinity of EST2-S35C toward paraoxon through a stable covalent complex has been previously demonstrated [19, 28].

A decrease in the fluorescence intensity of the immobilized biosensor was observed after paraoxon addition without major changes in the wavelength values (461–466 nm) of the maximum emission peak, in agreement with previous results [19]. Being the cysteine 35 binding the IAEDANS probe at the entrance of EST2 catalytic site [19], the fluorescence quenching we observed could be related to a structural rearrangement around the paraoxon molecules of the amino acid residues in the active site. This structural rearrangement, confirmed also by the tryptophan quenching [19], could increase structural local rigidity near the cysteine 35 and/or reduce its solvent exposure, affecting the fluorescence of IAEDANS. Further studies exploiting the crystallographic structures of EST2-S35C in presence and absence of paraoxon will help to better describe the fluorescence quenching observed.

**Fig. 4:**
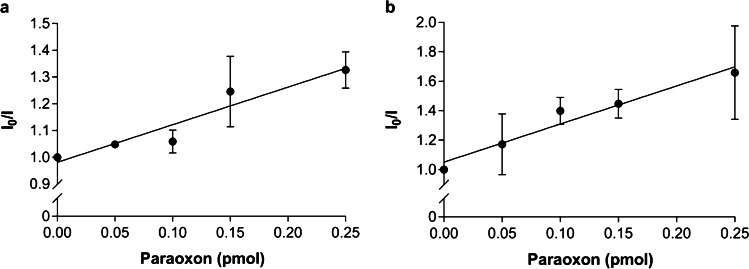
Ratios between the fluorescent intensity (mean ± SD, *n* = 3) at the maximum of emission (462–468 nm) in the absence (*I*_0_) and presence (*I*) of increasing amounts of paraoxon (pmol), using two different amounts of labelled EST2-S35C immobilized in the membrane: (a) 30 pmol; (b) 90 pmol

The ratio *I*_0_/*I* versus paraoxon amount (Fig. 4) gives the fluorescence quenching due to the quencher addition. The linear response after paraoxon addition was observed for both quantities of bioreceptor, 30 pmol (*Y* = 1.40*X* + 0.98, *r*^2^ = 0.91, *Sy.x* = 0.05, *F*_1,3_ = 30.33, *P* = 0.012, Fig. 4a) and 90 pmol (*Y* = 2.59*X* + 1.05, *r*^2^ = 0.95, *Sy.x* = 0.06, *F*_1,3_ = 60.92, *P* = 0.004, Fig. 4b). By measuring the differences in the intensity of fluorescence emission in the presence of a quencher and comparing it with a control signal, fluorescence techniques allow obtaining a biosensor with high sensitivity and monitoring the presence of very low amounts of the pesticide in the medium. In this study, we calculated a limit of detection (LOD) of 0.09 pmol and a limit of quantification (LOQ) of 0.31 pmol for paraoxon. These values are approximately one thousand times lower than previous detection levels obtained using the EST2-labelled bioreceptor in aqueous solution [18]. On the other hand, a similar LOD was achieved for paraoxon using fluorogenic substrate in a robotic workstation [20, 27].

**Fig. 5:**
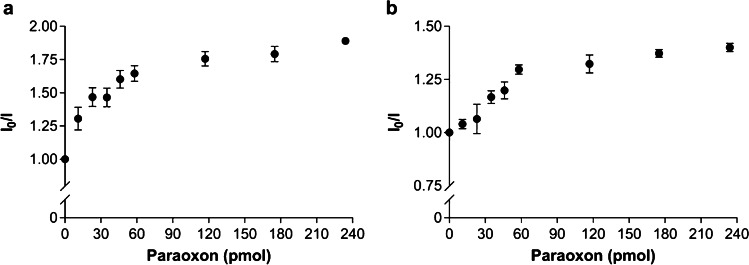
Ratios between the fluorescent intensity (mean ± SD, *n* = 3) at the maximum of emission (461–467 nm) in the absence (*I*_0_) and presence (*I*) of paraoxon with increasing amounts of paraoxon (pmol), using two different amounts of labelled EST2-S35C immobilized in the membrane: (a) 30 pmol; (b) 90 pmol

The amount of ligand influences the intensity of the measured fluorescence signal. So, it is possible to determine the pesticide concentration in the sample by a *I*_0_/*I* plotting also at higher concentrations (Fig. 5). A linear increase of *I*_0_/*I* would be expected until a 1:1 stoichiometric ratio inhibitor/enzyme. Nevertheless, the 30 pmol EST2-charged membranes showed a dynamic linear range up to 30 pmol of paraoxon. A plateau was indeed observed approximately at 60 pmol of paraoxon for the 90 pmol EST2-charged membranes. This behaviour may be ascribed to undesired fluorescence interference from adsorbed paraoxon at very high concentrations.

**Fig. 6:**
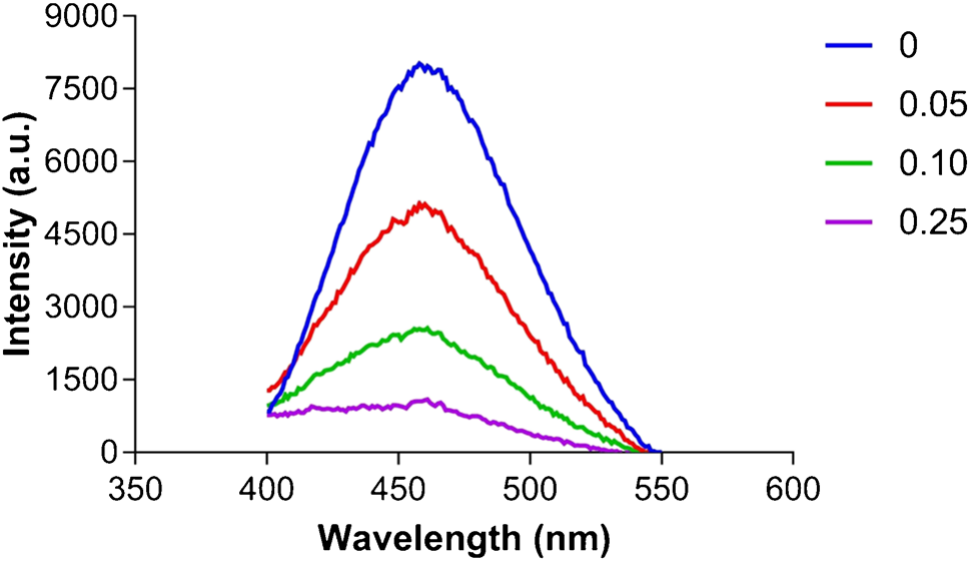
Fluorescence emission spectra after excitation at 340 nm of the 90 pmol immobilized labelled EST2-S35C under increased amounts of paraoxon (0, 0.05, 0.10, and 0.25 pmol), performed at the Horiba Fluoromax-4 spectrofluorometer

The measurements performed at the Horiba Fluoromax-4 spectrofluorometer with the respective adapted support for the membrane gave comparable results to the Jasco FP-8200 spectrofluorometer, showing high-intensity measurement at similar wavelength (461–462 nm) and fluorescence intensity decrease with increasing paraoxon amount (Fig. 6).

The 3D printed adapter and membrane supports showed good replicability and versatility, being used in different systems with minor adjustments in the membrane support.

We demonstrated that the designed 3D printed adapter and membrane supports can be used for fluorescence measurements and applied to detect chemicals in biosensing devices. The performance of the adapter was very high, reaching similar results of a robotic workstation in terms of sensitivity and replicability [20, 27]. Moreover, the data obtained for the fluorescence of the bioreceptor immobilized in the adapter also agrees with the previous data obtained in aqueous systems [18, 19].

### Comparison with other esterase-based biosensors

From the comparison between the developed methodology and previous biosensors for paraoxon detection (Table 1), the LOD value obtained using our EST2 enzyme is comparable to, or lower than, other LOD values measured using other esterase-based biosensors. Specifically, AChE-based methodologies presented a very low LOD due to the high specificity of AChE enzyme to paraoxon [41–50]; however, the use of EST2 allowed to achieve a low detection limit approaching the performance of AChE, and better sensitivity if compared to other enzymes, as is the case of the organophosphate hydrolase (OPH) used in two biosensing assays performed by Jain et al. [51]. Moreover, from the comparison with the reviewed literature, it emerges that our biosensor presents the lowest incubation time (1 min) compared to the other methodologies, and lower production cost being easily over-expressed and purified by a single thermo-precipitation step, increasing the performance efficiency in terms of time saving and costs. In addition, its high stability supports the use of EST2 in real applications.

**Table 1:** Comparison of esterase-based biosensors for the detection of paraoxon developed in the last 10 years

Enzyme	LOD (M)	Incubation time	Reference
EST2	9 × 10^−8^	1 min	This work
AChE	6.055 × 10^−14^	15 min	Li et al. 2015 [41]
	1.3 × 10^−13^	10 min	Peng et al. 2017 [42]
	3.4 × 10^−13^	30 min	Liang et al. 2020 [43]
	4 × 10^−12^	12 min	Mahmoudi et al. 2019 [44]
	1.4 × 10^−11^	10 min	Zhang et al. 2019 [45]
	1.8 × 10^−11^	Not specified	Chen et al. 2012 [46]
	6.5 × 10^−10^	10 min	Zhang et al. 2014 [47]
	7 × 10^−10^	5 min	Lang et al. 2016 [48]
	1 × 10^−9^	10 min	Mishra et al. 2012 [49]
	4 × 10^−9^	60 min	Dutta et al. 2014 [50]
Phosphotriesterase (OPH)	1.4 × 10^−5^ 3.9 × 10^−5^ (solution) 4.5 × 10^−5^ (encapsulating)	5 min 5 min (solution) 10 min (encapsulating)	Jain et al. 2021 [51]

## Conclusions

This work provides the advantage of introducing low-cost strategies for using ad hoc laboratory materials and overcoming the cost of commercial accessories. 3D printing gives the chance to produce new accessories in different scientific fields able to improve existing methodologies, or to design new ones. We designed a 3D adapter that is employable in different spectrofluorometers, having the fluorescence-cuvette dimension and the right angle to be used with minor light scattering. In addition, the developed membrane supports can be easily switched to match the specific heights of the instruments from different manufacturers.

In conclusion, the great advantage of using a 3D adapter for biosensing devices lies in the possibility of using immobilized enzymes on membranes, or direct measurement of optically active thin layers. The 3D support with the immobilized enzyme allows the washing of the membrane to remove the unreacted substances, including other organic compounds such as pigments, or amino acids, among others, improving the fluorescence measurements and decreasing the background noise.

## Supplementary Information

Below is the link to the electronic supplementary material.

Supplementary file1 (PDF 2269 kb)

## Data Availability

All .dae and .stl files described in this work are freely available under a creative commons license (https://www.thingiverse.com/febbraio-research-group/designs). Raw data are available upon request.
