# Supplementary material for: A 3D printable adapter for solid-state fluorescence measurements: the case of an immobilized enzymatic bioreceptor for organophosphate pesticides detection
Source: Anal Bioanal Chem. 2022 Jan 22;414(5):1999–2008. doi: 10.1007/s00216-021-03835-1 (PMC8791905; doi:10.1007/s00216-021-03835-1)

Supplementary Information

A 3D printable adapter for solid-state fluorescence measurements: the case of an immobilized enzymatic bioreceptor for organophosphate pesticides detection

Andreia C. M. Rodrigues1§, Maria Vittoria Barbieri1§, Marco Chino2*, Giuseppe Manco1$, Ferdinando Febbraio1$*

1 Institute of Biochemistry and Cell Biology - National Research Council (CNR). 80131 Naples, Italy.
2 Department of Chemical Sciences, University of Naples "Federico II". 80126 Napoli, Italy.

*Correspondence: Marco Chino - marco.chino@unina.it; Ferdinando Febbraio - ferdinando.febbraio@cnr.it.

§ These authors contributed equally to this manuscript (co-first authors)
$ These authors contributed equally to this manuscript (co-last authors)

Table S1. Maximum emission wavelength, fluorescence intensity (arbitrary units, n=3), and standard deviation in percentage of the EST2-S35C by means of the 45º and 30º adapters.

	45º			30º		
Sample	Wavelenght (nm)	S/N	% SD	Wavelenght (nm)	S/N	% SD
60 pmol EST2-S35C	460	2008.15	60.64	465	54694.59	9.76
	461	9188.84		466	45867.84	
	462	7030.21		466	46999.68	
90 pmol EST2-S35C	462	68273.94	31.12	466	711483.93	4.37
	467	47669.10		466	654103.84	
	466	37064.74		466	698315.33	
150 pmol EST2-S35C	466	170470.49	77.39	466	876830.24	3.66
	467	27915.53		466	883983.20	
	466	80923.12		466	937132.61	

Table S2. Maximum emission wavelength, fluorescence intensity (arbitrary units, n=3), and standard deviation in percentage of labelled EST2-S35C under different conditions, as a funcion of the membrane support

Membrane support	Sample	Wavelenght (nm)	Intensity	% SD
Support 1	150 pmol EST2-S35C	463	1048.96	22.01
bottom window height from base 15		462	732.73	
upper window heigth from base 21		462	727.63	
window height 6	150 pmol EST2-S35C + Px 5ul 30µM	462	727.07	2.83
		463	693.29	
		463	691.91	
	150 pmol EST2-S35C + Px 5ul 30µM	462	677.40	2.23
		464	653.01	
		462	650.87	
	150 pmol EST2-S35C + Px 5ul 30µM	462	635.94	5.43
		462	662.84	
		462	594.75	
	150 pmol EST2-S35C + Px 5ul 30µM	462	654.98	4.81
		462	624.82	
		462	594.82	
Support 2	150 pmol EST2-S35C	466	1674.56	6.98
bottom window height from base 13		465	1572.92	
upper window heigth from base 20		465	1455.96	
window height 7	150 pmol EST2-S35C + Px 5ul 30µM	464	1410.59	3.80
		465	1324.47	
		464	1319.21	
	150 pmol EST2-S35C + Px 5ul 30µM	465	1284.23	3.51
		464	1220.39	
		465	1201.48	
	150 pmol EST2-S35C + Px 5ul 30µM	464	1176.88	2.16
		464	1170.88	
		464	1130.87	
	150 pmol EST2-S35C + Px 5ul 30µM	464	1078.28	07.06
		466	940.33	
		464	1045.85	
	150 pmol EST2-S35C + Px 5ul 30µM	464	1043.77	0.87
		464	1038.93	
		464	1026.36	

SD: standard deviation

Table S3. Maximum emission wavelength, fluorescence intensity (arbitrary units, n=3), and standard deviation in percentage as a function of labelled EST2-

Sample	Amount of protein (pmol)	Wavelenght (nm)	Intensity	% SD
0.5 ul EST2-S35C	15	473	530.97	54.61
		473	1488.63	
		474	743.26	
1 ul EST2-S35C	30	473	522.17	18.70
		477	384.39	
		472	381.74	
2 ul EST2-S35C	60	473	2495.88	32.85
		474	4953.67	
		473	3777.37	
4 ul EST2-S35C	120	465	2624.58	5.67
		467	2621.11	
		466	2373.43	
6 ul EST2-S35C	180	467	2426.38	1.70
		465	2510.01	
		467	2472.49	

Fig. S1 Mechanism of the inhibition of EST2 by diethyl (4-nitrophenyl) phosphate (paraoxon). After the paraoxon enters the catalytic site, the reactive serine 155 bind covalently to the phosphate atom, while the histidine 282 share a hydrogen atom with the oxygen, mediating the release of the 4-nitrophenol.

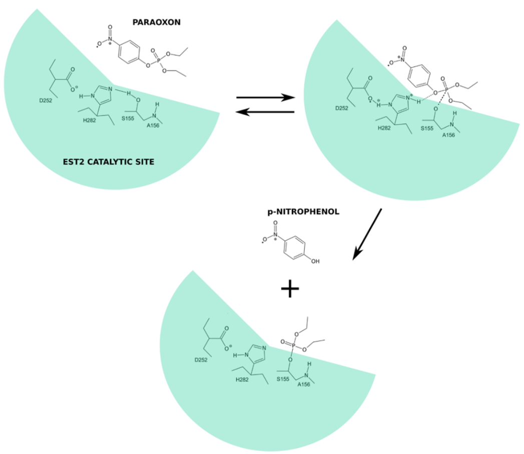

Fig. S2 Holder geometry prevents noise from incoming wavelength: a) light geometry scheme, b) Printed holder and support in the Jasco Fluorometer.

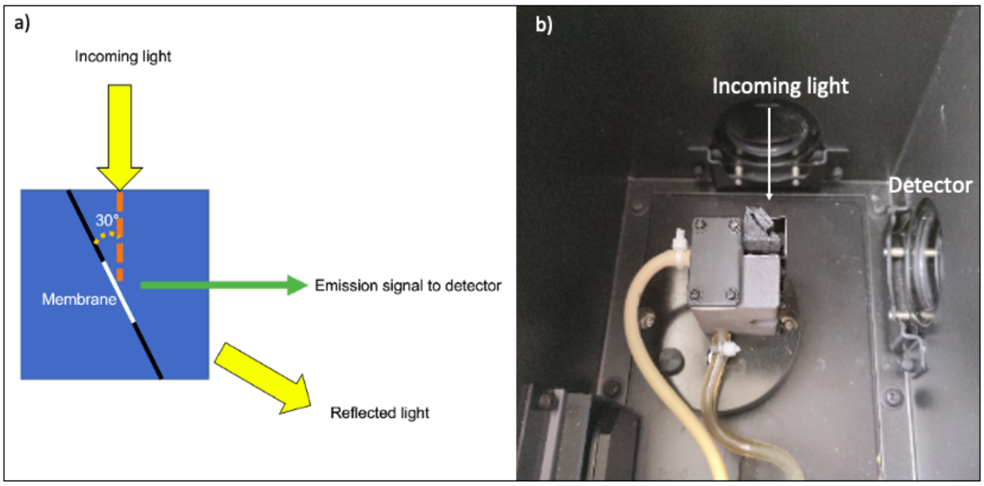

Fig. S3 Commercially available solid-sample holder for Horiba Fluoromax-4 spectrofluorometer: a) solid-sample holder; b) mounted holder into the Fluoromax-4.

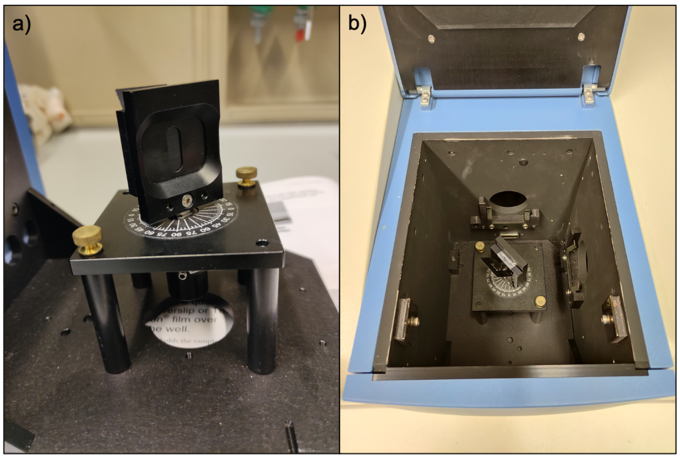

Fig. S4 Spectra obtained for the reading of a membrane containing 90pmol of labelled EST2-S35C in the commercial membrane holder of HORIBA Fluoromax-4 spectrofluorometer.

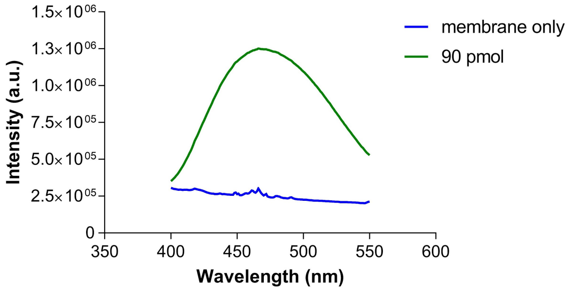

Supplement: Supplementary file 1 — Supplementary file1 (PDF 2269 kb) [file 216_2021_3835_MOESM1_ESM.pdf]
